# Association Study Reveals Novel Genes Related to Yield and Quality of Fruit in Cape Gooseberry (*Physalis peruviana* L.)

**DOI:** 10.3389/fpls.2018.00362

**Published:** 2018-03-20

**Authors:** Francy L. García-Arias, Jaime A. Osorio-Guarín, Victor M. Núñez Zarantes

**Affiliations:** Corporación Colombiana de Investigación Agropecuaria, Corpoica, Centro de Investigación Tibaitatá, Mosquera, Colombia

**Keywords:** GWAS, fruit traits, *Physalis peruviana*, mixed linear model, SNP markers

## Abstract

Association mapping has been proposed as an efficient approach to assist plant breeding programs to investigate the genetic basis of agronomic traits. In this study, we evaluated 18 traits related to yield, (FWP, NF, FWI, and FWII), fruit size-shape (FP, FA, MW, WMH, MH, HMW, DI, FSI, FSII, OVO, OBO), and fruit quality (FIR, CF, and SST), in a diverse collection of 100 accessions of *Physalis peruviana* including wild, landraces, and anther culture derived lines. We identified seven accessions with suitable traits: fruit weight per plant (FWP) > 7,000 g/plant and cracked fruits (CF) < 4%, to be used as parents in cape gooseberry breeding program. In addition, the accessions were also characterized using Genotyping By Sequencing (GBS). We discovered 27,982 and 36,142 informative SNP markers based on the alignment against the two cape gooseberry references transcriptomes. Besides, 30,344 SNPs were identified based on alignment to the tomato reference genome. Genetic structure analysis showed that the population could be divided into two or three sub-groups, corresponding to landraces-anther culture and wild accessions for *K* = 2 and wild, landraces, and anther culture plants for *K* = 3. Association analysis was carried out using a Mixed Linear Model (MLM) and 34 SNP markers were significantly associated. These results reveal the basis of the genetic control of important agronomic traits and may facilitate marker-based breeding in *P. peruviana*.

## Introduction

*Physalis peruviana* L is also known as cape gooseberry, golden berry, ground cherry, rasbhari, and winter cherry in different parts of the world. It is an exotic fruit that belongs to the Solanaceae family and is well-known for its nutritional value (high contents of vitamins A, C, and B), micronutrient content (phosphorus, calcium, and iron), and antioxidant, anti-inflammatory, and anti-hepatotoxic activities (Wu et al., 2006; Ramadan, 2011; Ramadan et al., 2015). *P. peruviana* fruits are desirable for confections, dried-fruit snacks, and fresh consumption. Colombia is the world's top producer of this fruit, followed by South Africa (Bonilla et al., 2009). It is the second most exported fruit from Colombia, trailing only the banana. In 2014, 13,260 tons were harvested, mainly from the departments Boyacá, Antioquia, and Cundinamarca. Exports to the Netherlands, Germany, Belgium, and Canada accounted for 5,852 tons with sales of 30 million dollars (Agronet, 2016). Despite great promise, production yield decreased from 13.76 t/ha in 2010 to 9.81 t/ha in 2014 (Agronet, 2016) partly as a result of the vascular wilt disease caused by *Fusarium oxysporum* (Cotes et al., 2012). Additionally, 20–45% of harvested fruits are discarded because of cracking problems (Fischer, 2005) and 15% of the fruit production does not satisfy the standards of size and quality required for export (Valdenegro et al., 2013), significantly reducing the volume of exportable fruit.

There is a desire to develop varieties with high fruit yield and quality, especially those with resistance to cracking and that meet the standards of the size required for the market. Given the success in related species such as tomato (*Solanum lycopersicum* L.), where crack-resistant material has been developed (Matas et al., 2004), it seems likely that similar improvement may be expected for the cape gooseberry. Developing resources and increasing genetic knowledge on fruit quality and yield characteristics in the cape gooseberry will accelerate the time of development of new varieties, facilitating the identity of the cultivar, the evaluation of genetic diversity, the selection of parents, and the confirmation of hybrids with the use of Marker Assisted Selection (MAS) (Chhetri et al., 2017; Favoretto et al., 2017).

The identification of SNP markers responsible for natural phenotypic variation may be detected with Association Mapping (AM) (Soto-cerda and Cloutier, 2012; Xu et al., 2017). AM is a strategy based on Linkage Disequilibrium (LD) that ultimately seeks to identify specific functional variants linked to phenotypic differences in a particular trait. The polymorphisms in the DNA sequence responsible for phenotypic change can be detected and then introgressed into crop germplasm (Flint-Garcia et al., 2003; Oraguzie et al., 2003; Abe et al., 2012; Xu et al., 2017). AM uses unstructured populations for trait mapping based on the strength of high-throughput genotyping and phenotypic characterization. Genotyping By Sequencing (GBS) has allowed high-throughput identification of molecular markers in the rose (Heo et al., 2017), apple (Norelli et al., 2017), pepper (Taranto et al., 2016), and pigeonpea (Saxena et al., 2017) at low costs (Voss-fels and Snowdon, 2016). The success of GBS in maize (Elshire et al., 2011), potato (Uitdewilligen et al., 2013), sesame (Uncu et al., 2016), and wheat (Poland et al., 2012; Kobayashi et al., 2016) suggest a role for the technique in non-model specialty crops. This technique is based on the reduction of genome complexity through methylation-sensitive restriction enzymes (Elshire et al., 2011), making it possible to search for polymorphisms in species with large genomes, high diversity, or without a reference genome (Poland and Rife, 2012). The utility of GBS in the cape gooseberry, a species without a reference genome, has previously been demonstrated in the identification of candidate genes associated with the resistance response to *F. oxysporum* (Osorio-Guarín et al., 2016), using tomato (*S. lycopersicum*) as a reference genome for the SNP calling process.

The phylogeny reconstruction carried out by Garzón-Martínez et al. (2012) demonstrated a close relation between *P. peruviana* and *S. lycopersicum*. The tomato is a diploid species with a haploid set of 12 chromosomes and a small genome (950 Mb), encoding ~35,000 genes that are sequestered mainly in the adjacent euchromatic region (Barone et al., 2008). In contrast, the cape gooseberry has a chromosomic complement of 2*n* = 4x = 48 and a large genome size ranging from 1410.77 to 1985.34 Mb (Liberato et al., 2015). Despite considerable differences in genome size, the comparative analysis conducted in Solanaceae family by Wang et al. (2008) revealed high-degree sequence synteny in chromosomal regions with small-scale differences between species, as a result of nucleotide substitutions, insertions, deletions, tandem duplications of individual genes, inversions, and transpositions. Therefore, tomato genes could be conserved in the cape gooseberry.

GBS is a useful approach for analyze the genetic diversity, population structure, and offers an ultimate MAS tool to accelerate plant breeding. This is the first time that GBS methodology was implemented in cape gooseberry for mapping SNP markers of fruit quality traits. The objectives of this study were to: (1) evaluate 18 phenotypic traits of 100 cape gooseberry accessions from the Corporación Colombiana de Investigación Agropecuaria—Corpoica germplasm collection; (2) examine the level of genetic diversity and the population structure within the cape gooseberry collection; and (3) identify candidate genes/SNPs significantly associated with yield, fruit size, and quality.

## Materials and methods

### Plant material and experimental design

Cape gooseberry accessions were selected from the germplasm collection maintained at the Corporación Colombiana de Investigación Agropecuaria—Corpoica. The collection consisted of 100 accessions based on 77 accessions reported by Osorio-Guarín et al. (2016) and 23 new accessions derived from anther culture germplasm that included doubled haploids and haploids accessions (Table S1).

The plantlets were propagated clonally by *in vitro* subculturing of node cuttings at 4–6 weeks using Murashige and Skoog (MS) medium supplemented with 0.1 mg L^−1^ GA_3_. The plantlets were acclimated in a chamber with 80% of relative humidity. After acclimation, those plantlets were cultivated using a triple lattice experimental design at the Corpoica Tibaitatá research center (4°40′55.5″N, 74°12′12.4″). The accessions were grown in three replicates using a row-to plant and plant to plant spacing of 2 m. Three plants per accession were grown per replication. The Kenyan and Colombian ecotypes were used as reference accessions (Peña et al., 2011; Criollo et al., 2014).

### Phenotyping

A total of 18 traits evaluated were distributed into three categories: yield, fruit size-shape, and fruit quality (Tables 1, 2). For the yield category: Fruits Weight per Plant (FWP, weight of fruits during all harvests), Plant Fruit Number (NF, the number of fruits during all harvests), and Fruit Weight with and without calyx traits (FWI-FWII, mean weight of 10 fruits in each harvest) were evaluated in eight harvests.

**Table 1:** Analysis of variance for 18 traits of cape gooseberry accessions.

**Category**	**Trait**	**Mean square**		
		**Replications**	**Treatments**	**Error**
Yield	Fruits weight per plant (FWP)	14450419.5^*^	1113325.7^*^	373440.7
	Number of fruits (NF)	575128.1^*^	26941.3^*^	11127.7
	Fruit weight with calyx (FWI)	6.4^*^	9.3^*^	0.3
	Fruit weight without calyx (FWII)	1.8^*^	6.7^*^	0.2
Fruit size-shape	Fruit perimeter (FP)	2.1^*^	2.7^*^	0.14
	Fruit area (FA)	1.3^*^	1.94^*^	0.10
	Fruit maximum width (MW)	0.14^*^	0.27^*^	0.01
	Fruit width Mid-height (WMH)	0.1^*^	0.27^*^	0.01
	Fruit maximum height (MH)	0.11^*^	0.25^*^	0.01
	Fruit height mid-width (HMW)	0.11^*^	0.28^*^	0.01
	Distal end indentation area (DI)	0.0000003	0.004^*^	0.00002
	Fruit shape index external I (FSI)	0.0005	0.007^*^	0.001
	Fruit shape index external II (FSII)	0.0007	0.01^*^	0.001
	Asymmetry ovoid (OVO)	0.0002	0.006^*^	0.0002
	Asymmetry obovoid (OBO)	0.0001	0.004^*^	0.00004
Fruit quality	Cracked fruits percentage (CF)	11.89	345.3^*^	30.5
	Firmness (FIR)	0.02	0.2^*^	0.02
	Soluble solids concentration (SST)	9.5^*^	1.5^*^	0.27

* *indicate significant at p < 0.0001 level*.

**Table 2:** Phenotypic variation of 18 traits analyzed in the complete collection.

**Category**	**Trait**	**Units**	**Minimum**	**Maximum**	**Mean**	**CV**	**${\text{h}}_{\text{bs}}^{2}$ (%)**
Yield	Fruits weight per plant (FWP)	g/plant	22.44	8, 304.51	3, 863.45	47.45	66.46
	Number of fruits (NF)	N°/plant	81.94	1, 471.79	685.25	46.18	58.70
	Weight of fruits with calyx (FWI)	g/fruit	0.26	10.69	5.75	9.63	96.77
	Weight of fruits without calyx (FWII)	g/fruit	0.15	9.24	4.73	10.54	97.01
Fruit size-shape	Fruit perimeter (FP)	Cm	2.98	8.87	6.85	5.42	94.81
	Fruit area (FA)	cm^2^	0.55	5.20	3.28	9.75	94.85
	Fruit maximum width (MW)	Cm	0.96	2.81	2.07	5.56	96.30
	Fruit width Mid-height (WMH)	Cm	0.93	2.78	2.06	5.54	96.30
	Fruit maximum height (MH)	Cm	0.76	2.3	1.93	4.50	96.00
	Fruit height mid-width (HMW)	Cm	0.63	2.25	1.90	4.51	96.43
	Distal end indentation area (DI)	cm^2^	0	0.18	0.0092	42.65	99.50
	Fruit shape index external I (FSI)	–	0.70	1.06	0.93	3.17	85.71
	Fruit shape index external II (FSII)	–	0.68	1.06	0.92	3.56	90.00
	Asymmetry ovoid (OVO)	–	0	0.21	0.15	9.51	96.67
	Asymmetry obovoid (OBO)	–	0	0.18	0.0093	71.34	99.00
Fruit quality	Cracked fruits percentage (CF)	%	0	59.27	6.34	88.66	91.17
	Firmness (FIR)	lb-f	0.69	2.23	1.67	7.53	90.00
	Soluble solids concentration (SST)	°Brix	13.12	17.27	14.85	3.47	82.00

*Minimum, maximum, mean values, coefficient of variation (CV), and broad-sense heritability (${\text{h}}_{\text{bs}}^{2}$)*.

For fruit size-shape category, nine fruits from each accession per replicate were evaluated and cut longitudinally through the center, placed cut-side down on a Hewlett Packard® C9866A and digitalized at 200 dots per inch. The Tomato Analyzer software v3.0 (Rodríguez et al., 2010) was used to measure: Fruit Perimeter (FP), Fruit Area (FA), Width at Mid Height (WMH, the width measured at ½ of the fruit's height), Maximum Width (MW, the maximum horizontal distance of the fruit), Height at Mid Width (HMW, the height measured at ½ of the fruit's width), and Maximum Height (MH, the maximum vertical distance of the fruit). For fruit shape, the following traits were measured: Distal end Indentation Area (DI, distal end indentation area relative to total fruit area), the Fruit Shape Index External I (FSI, the ratio of the maximum height to the maximum width), the Fruit Shape Index external II (FSII, the ratio of height mid-width to width mid-height), and the Asymmetry As Ovoid (OVO) when the area of the fruit is higher above mid-height than below it or Obovoid (OBO) when the area of the fruit is greater below mid-height than above it.

For fruit quality category, measurements were assessed in eight harvests except for firmness which was evaluated in six harvests in the population. The fruit was collected at the optimal harvest point corresponding to maturity stage 3 as determined by the color according to the NTC 4580 Standard (Instituto Colombiano de Normas Técnicas-Icontec, 1999). The percentage of Cracked Fruits (CF) per plant was determined based on the average of cracked fruits in all harvests and the Firmness (FIR) was measured using Chatillon TDC200 Digital Force Tester. Soluble Solids Concentration (SST) was measured as °Brix with a hand-held refractometer ATAGO PAL1 on a minimum of 3 mature fruits per accession per replicate.

### Statistical analysis of phenotypic data

Analysis of variance (ANOVA) was performed using the General Linear Model (GLM) procedure in SAS software (SAS Institute, Cary, NC) to determine the existence of significant differences between accessions for the quantitative traits evaluated. The model tested was Y = G + Rep + Error, with all factors considered fixed. Principal Component Analysis (PCA) and the Cluster Analysis (CA) by the Ward method (semi-partial *R*^2^ = 0.10) were also conducted with SAS software. Correlations among traits, were detected using Pearson's correlation coefficient (*r*) at *P* = 0.05. Broad-sense heritability (${\text{h}}_{\text{bs}}^{2}$) of all traits was calculated using the formula as described by Allard (1960) as follow: ${\text{h}}_{\text{bs}}^{2}=$ [(σ^2^G)/(σ^2^P)] × 100, where: σ^2^G = Genotypic variance; σ^2^P = Phenotypic variance.

### Genotyping and SNP markers calling

Genomic DNA of 77 accessions was previously isolated by Osorio-Guarín et al. (2016). The DNA of the 23 new accessions was isolated from 100 mg of leaf tissue collected from *in vitro* grown plants. Tissue was macerated in liquid nitrogen using a mortar and pestle. DNA extraction was performed using the DNeasy Plant Mini Kit (Qiagen, Valencia, USA) according to the manufacturer's protocol. Total DNA was quantified by NanoDrop 1000 spectrophotometer (NanoDrop Technologies, Wilmington, DE, USA), and the quality was checked through restriction enzyme digestion with HindIII enzyme and visualized by electrophoresis using 2% agarose gels. The GBS libraries were constructed using the restriction enzyme ApekI (GCWGC) and sequenced twice with the Illumina HiSeq (Illumina Inc. San Diego, CA) next-generation sequencing platform at the Cornell Genomic Diversity Facility.

SNP calling was performed using the Tassel-GBS pipeline v5.0 (Bradbury et al., 2007). A filtered HapMap was created with the following parameters: minimum minor allele frequency (mnMAF) of 0.05, minimum locus coverage (mnLCov) of 0.8, minimum taxon coverage (mnTCov) of 0.3, and minimum site coverage (mnSCov) of 0.7.

### Genetic diversity and population structure

Standard measures of diversity including Expected Heterozygosity (H_E_), Observed Heterozygosity (H_O_), and Polymorphic Information Content (PIC) were calculated by PowerMarker v3.2 (Liu and Muse, 2005) using filtered SNP markers. The alignment was realized with Bowtie2 (Langmead and Salzberg, 2012) against the two cape gooseberry references transcriptomes (leaf and root NCBI Bioproject: PRJNA67621) and the tomato reference genome version SL2.40.

Population structure analysis was carried out based on software Admixture v1.3.0 (Alexander and Novembre, 2009) in an unsupervised mode. This program estimates individual admixture proportions from multi-locus SNP data using a maximum-likelihood method. It employs a similar statistical model as the program Structure (Pritchard et al., 2000) but uses fast numerical optimization algorithm to achieve greater speed. This computational efficiency provides an advantage mainly when using very large numbers of markers and individuals (Liu et al., 2013). The number of populations (K) was set from 1 to 10 and the K optimum was selected based on the cross-validation error compared to other K values. The Q estimates (Q matrix) of the K optimum was used to the association mapping and was plotted in R software (R Team, 2014). To corroborate the population structure, the Neighbor-Joining algorithm was used for cluster analyses based on the Nei's genetic distance. Three-dimensional scatter plot was carried out with the results of the Principal Component Analysis (PCA) which was performed using Tassel v5.0 based on N x SNP matrix (Bradbury et al., 2007).

### Association analysis

Linkage disequilibrium for each marker pair was calculated using *r*^2^ parameter with sliding windows size of 50 sites (bp) through the software Tassel v5.0 (Bradbury et al., 2007). Associations between molecular markers and phenotypic data were computed using the Genome Association and Prediction Integrated Tool—GAPIT (Lipka et al., 2012) based on the Mixed Linear Model (MLM) that controls the population structure and genetic relatedness among the individuals by incorporating the Q and K matrices. The kinship coefficients (K matrix) between individuals were estimated according to the method of Loiselle (Loiselle et al., 1995). For the association analysis, the non-normal dataset of phenotypic traits was transformed with Box-Cox transformation procedure using the software Statistica v12.0 (Statsoft Inc., Tulsa, USA). The *p*-values were adjusted with multiple testing, according to Benjamini and Hochberg (1995), to control the False Discovery Rate (FDR). The amount of phenotypic variation explained by each marker was estimated by *r*^2^. Associations were considered significant when *p* ≤ 0.0001 or LOD scores greater than 4.0. Finally, the biological function of the associated markers was identified in JBrowse environment from Sol Genomics Network (SGN) (Fernandez-Pozo et al., 2015) using tomato genome version SL2.40 and the ITAG annotation version 2.4.

## Results

### Phenotyping

The traits related to yield, fruit size-shape, and fruit quality categories showed a high coefficient of variation (CV), suggesting both phenotypic variations in the germplasm and representativeness of the gene pool. The mean squares values from the ANOVA of the 18 quantitative traits for the 100 accessions showed highly significant differences (*p* ≤ 0.0001) for all of the studied characteristics (Table 1). In contrast, there were no significant differences between the replications for the DI, FSI, FSII, OVO, OBO, CF, and FIR traits. The variability for the more important traits measured as %CV ranged from 3.47 to 10.54%, and the traits DI, NF, FWP, OBO, and CF exhibited higher levels of variation with 42.65, 46.18, 47.45, 71.34, and 88.66%, respectively (Table 2).

The yield traits presented moderate to high variation and ranged from 9.63 (FWI) to 47.45 (FWP). The fruit weight per plant (FWP) ranged between 22.44 and 8304.51 g per plant, the number of fruits (NF) ranged between 81.94 to 1471.79 fruits per plant, and the weight of fruits with and without calyx ranged between 0.15 to 0.26 and 9.24 to 10.69 g per fruit, respectively (Table 2). The accessions 09U033_1 and 09U277_5 showed a FWP greater than 7,500 g/plant, higher than the Kenyan and Colombian ecotypes, considered as references (Table S2). For the FWI and FWII traits, the haploid accessions 12U398_1, 09U294_6, 12U366_1, 14U447_1, 14U425_1, and 09U295_4 represented outlier values since these accessions exhibited a value less than 0.26 g, as compared to a mean value of 4.73 g for the entire population. In contrast, the accessions 09U134_3, 14U426_1, and 14U426_2 exhibited a value greater than 7.8 g, the first one corresponded to an accession that originated from Nepal and the last two correspond to Kenyan accessions. The accessions that showed the highest NF were 09U033_1 and 09U277_5, both of which exhibited more than 1,400 fruits, while the accessions 14U449_1 and 12U398_1 showed less than 105 fruits. The Pearson correlation coefficients (*r*) among the yield traits showed a positive value for FWP, with NF (*r* = 0.75), FWI (*r* = 0.59), and FWII (*r* = 0.58) having significant *p*-values > 0.0001 (Table S3).

The fruit size characteristics showed moderate variation ranging between 4.50% (MH) and 9.75% (FA) (Table 2). The WMH and HMW showed similar values to the WH and MH, respectively, suggesting that the traits did not provide additional relevant information. The accessions 09U134_3 and 09U282_3 showed extreme values for these traits, exhibiting a width and height greater than 2.5 and 2.2 cm, respectively (Table S2). In contrast, the haploid accessions presented the lowest values, less than 1.0 and 1.3 cm for width and height, respectively. The Pearson coefficient showed a high positive correlation for fruit perimeter (FP) with FA, WMH, MW, HMW, and MH (*r* ≥ 0.96). Similarly, the FP showed high positive correlations with FWI-FWII (*r* = 0.90) and a moderate positive correlation with FWP (*r* = 0.49).

The fruit shape traits showed moderate variation and most of the traits ranged between 3.17% (FSI) and 9.51% (OVO), but the DI and OBO showed 42.65 and 71.34% CV (Table 2). The FSI and FSII ranged from 0.68 to 1.06, indicating that the fruits showed a shape from round to elongated. Only the fruits of the haploid accessions exhibited a strong indentation area, exceeding 0.13 cm^2^, while accession 14U449_1 showed a low indentation area of 0.04 cm^2^ (Table S2). The obovoid asymmetry was found in 6% of the accessions, corresponding to haploid accessions with FSI-FSII < 0.8; while the ovoid asymmetry was found in 94% of the accession with FSI-FSII > 0.8. An exception was accession 14U449_1 with ovoid asymmetry demonstrated by FSI-FSII < 0.8 characteristics of an oblate shape. The Pearson coefficient showed a high negative correlation of OVO with DI and OBO (*r* ≥ 0.88) and moderate positive correlation with FSII (*r* = 0.53). Likewise, OVO showed high positive correlation with FWI-FWII (*r* = 0.77) and the fruit size traits: FP, FA, WMH, MW, HMW, and MH (*r* ≥ 0.82); moderate positive correlation with FIR (*r* = 0.67) and high negative correlation with CF (*r* = −0.71) (Table S3).

For the fruit quality traits, the percentage of cracked fruits (CF) showed the highest variation and ranged from 0 to 59.27% (Table 2). The accessions 09U026_1, 09U187_4, 09U131_3, 14U426_1, and 09U280_3 were uniform and exhibited values less than 0.2%, while accessions derived from anther culture exhibited high variation, generally greater than 37% (Table S2). The haploid accessions exhibited low firmness with less than 1.2 lb-f, an unsurprising result since this type of fruit has no seeds and no flesh at all. The accessions 09U026_1, 09U187_4, 09U131_3, 14U426_1, and 09U280_3 showed low CF and firmness greater than 1.5 lb-f. The trait SST showed the lowest variation and ranged from 13.13 to 17.27 °Brix. The accessions 12U398_1, 09U130_2, 14U420_1, 12U350_1, and 12U347_1 showed more than 16 °Brix and most of them were generated by anther culture technology, except accession 09U130_2. In contrast, the wild accession 09U193_1 showed only 13.1 °Brix. The FIR was negatively correlated with CF (*r* = −0.70), and had a moderate positive correlation with the yield traits: FWP, FWI, and FWII (*r* = 0.54), size of fruit traits: FP, FA, WMH, MW, HMW, and MH (*r* ≥ 0.57) and a positive correlated with OVO (*r* = 0.67) (Table S3). The broad-sense heritability (${\text{h}}_{\text{bs}}^{2}$) of the traits ranged from 58.70% for NF to 99.50% for DI.

Based on the PCA, the first four principal components had eigenvalues >1 and contributed 89.87% of the total cumulative variability among the different accessions. The first principal component (PC1), was represented mainly by the fruit weight and size traits (FWI, FWII, FP, FA, MW, WMH, MH, and HMW); fruit cracking and firmness explained ~63% of the observed variation and could be useful for selection schemes in cape gooseberry breeding. The PC2, represented by the number of fruits (NF) and fruit shape index I (FSI), explained 12.6% of the observed variation; whereas, PC3, primarily the fruit yield per plant (FWP) and fruit shape index I (FSII), explained 9% of the observed variation. The soluble solids concentration contributed strongly to PC4 and explained 5.3% (Table 3).

**Table 3:** Eigenvectors and eigenvalues for the first four principal components (PCs).

**Traits**	**PC1**	**PC2**	**PC3**	**PC4**
Fruits weight per plant (FWP)	0.189	0.324	**0.415**	0.003
Number of fruits (NF)	0.027	**0.504**	0.412	−0.150
Weight of fruits with calyx (FWI)	**0.265**	−0.123	0.141	−0.002
Weight of fruits without calyx (FWII)	**0.265**	−0.125	0.137	−0.036
Fruit perimeter (FP)	**0.274**	−0.158	−0.002	−0.035
Fruit area (FA)	**0.272**	−0.165	0.074	−0.048
Fruit maximum width (MW)	**0.270**	−0.209	0.080	−0.020
Fruit width Mid-height (WMH)	**0.271**	−0.204	0.084	−0.024
Fruit maximum height (MH)	**0.283**	−0.050	−0.058	−0.043
Fruit height mid-width (HMW)	**0.284**	−0.036	−0.074	−0.035
Distal end indentation area (DI)	−**0.259**	−0.012	0.206	−0.098
Fruit shape index external I (FSI)	0.090	**0.445**	−0.418	−0.122
Fruit shape index external II (FSII)	0.165	0.367	−**0.424**	−0.068
Asymmetry ovoid (OVO)	**0.263**	−0.027	−0.071	0.116
Asymmetry obovoid (OBO)	−**0.256**	0.016	0.202	−0.099
Cracked fruits percentage (CF)	−**0.217**	−0.157	0.0615	−0.022
Firmness (FIR)	**0.209**	0.185	−0.082	0.033
Soluble solids concentration (SST)	0.009	0.134	0.060	**0.955**
Eigenvalue	11.95	2.39	1.72	1.01
% of total variance	0.629	0.126	0.090	0.053
Cumulative variance	0.629	0.755	0.846	**0.899**

*The most contributing variables shown in bold*.

**Figure 1:**
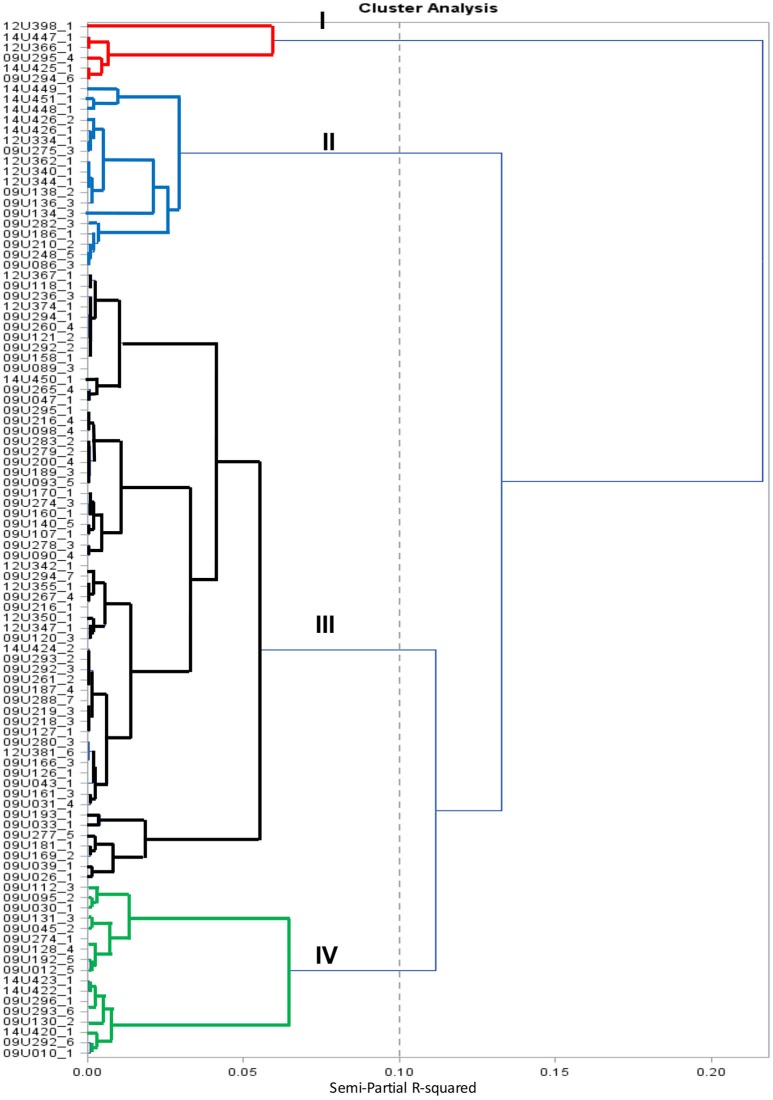
Cluster dendrogram of cape gooseberry collection using 18 phenotypic traits based on Ward method.

The cluster analysis grouped 100 cape gooseberry accessions into four groups, as shown in Figure 1. Group-I was comprised of 6 accessions, followed by 18, 59, and 17 accessions respectively in group-II, III, and group-V. The accessions in group-I presented small fruits (mean 0.80 g), low fruit firmness (mean 0.97 lb-f), and high fruit cracking (mean 36.88%), as compared to all other groups and it was represented by haploid accessions. Additionally, this group presented distal indentation and obovoid asymmetry. The second group (II) presented the largest fruits (mean 6.69 g), moderate fruit firmness and cracking (mean 1.57 lb-f and 9.15%, respectively). The fruit of these accessions had an ovoid shape and were collected mainly from Boyacá, Cundinamarca, and Nariño in Colombia and from international repositories in Denmark, France, Nepal, and Ecuador. The third group (III) consisted of 59 landraces from Antioquia, Boyacá, Caldas, Cundinamarca, Nariño, and Valle and was divided into two sub-groups. The first sub-group of accessions was derived from anther culture or collected from South Africa. These accessions presented intermediate-high fruit weight (mean 6.29 g), moderate fruit firmness, low fruit cracking (mean 1.75 lb-f and 2.55%, respectively) and an ovoid fruit shape. The second sub-group had seven accessions from Cundinamarca, Nariño, Boyacá, and Valle in Colombia, showed the lowest cracking percentage (<4%) with a production higher than 7,000 g/plant and are likely to be useful in cape gooseberry breeding programs. The fourth group (IV) presented an intermediate fruit weight (mean 4.61 g), moderate fruit firmness (mean 1.69 lb-f), low fruit cracking (mean 5.73%) and an ovoid fruit shape. These accessions are from Antioquia, Boyacá, Cundinamarca, Nariño, Norte de Santander, and Valle.

### Genotyping and population structure

A total of 225,161,229 reads were obtained with an average sequence length of 101 bp and phred quality score > 26. After filtering, we excluded accession 09U039-1 because of low-quality data. We identified 27,982, 36,142, and 30,344 SNPs for cape gooseberry leaf and root transcriptomes and the tomato genome had <4.4% of missing data (Table 4). The heterozygosity observed was found to be high with H_O_ = 0.725, and the average of H_E_ was 0.44, determined according to Nei (1973). The PIC, an estimate of the relative informativeness of each genetic marker, averaged 0.342.

**Table 4:** Genetic variability estimates of cape gooseberry collection.

**Transcriptomes/genome reference**	**SNPs default parameters**	**Filtered SNPs**	**H_O_**	**H_E_**	**PIC**
Leaf cape gooseberry transcriptome	45,184	27,982	0.731	0.447	0.343
Root cape gooseberry transcriptome	58,887	36,142	0.733	0.449	0.344
Tomato genome	50,805	30,344	0.711	0.439	0.338

**Figure 2:**
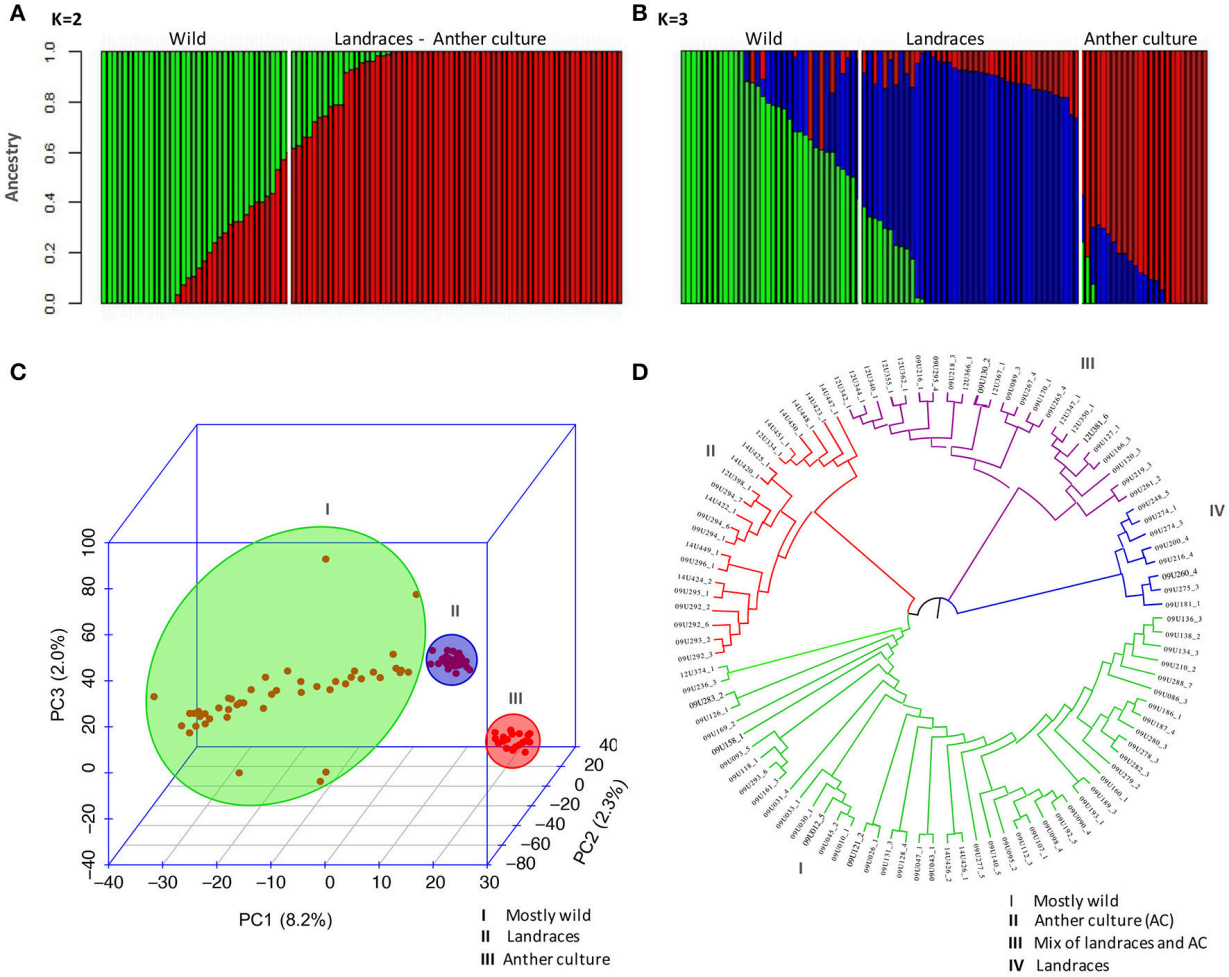
Population structure of cape gooseberry collection based on 30,344 SNPs markers. Inferred population structure of the cape gooseberry collection using the tomato SNPs matrix. Bar plot for *K* = 2 **(A)** and *K* = 3 **(B)** grouped by state of cultivation and the bar length represent the membership probability of each accessions belonging to different sub-populations. **(C)** Scatterplot of Principal Component Analysis scores of components PC1, PC2 and PC3 based on 30,344 SNP markers. **(D)** NJ-based dendrogram with cape gooseberry SNPs clustered into four sub-populations. Colors correspond to each sub-population which consisted of: mostly wild accessions (I-green), mostly AC accessions (II-red), mix of CA accessions and landraces (III-violet), and only landraces (IV-red).

The genetic structure of the entire population was assessed using the Admixture software and PCA. The results are presented for analysis using the set of 30,344 polymorphic SNP markers identified with the tomato genome as a reference because of the similar results of genetic diversity of this set when compared with the cape gooseberry transcriptomes. Additionally, with this strategy, one better understands the function of the associated markers because many tomato coding genes are well-reported and annotated with their biological functions. The optimal K of the population, inferred according to the cross-validation error, indicated that *K* = 2 and *K* = 3 can be the best number of sub-populations (Figure S1). For *K* = 2 (Figure 2A), the accessions were sub-divided into wild and a second sub-population that included landraces and anther culture accessions. The wild sub-population consisted of 33 accessions from the Colombian departments Antioquia, Boyacá, Nariño, Valle del Cauca, and international repositories in Denmark, Ecuador, France, Nepal, and South Africa. The second sub-population consisted of 66 accessions, most of which were landraces from the Colombian departments Antioquia, Boyacá, Caldas, Cundinamarca, Nariño, and Norte de Santander. Additionally, the accessions obtained with the *in vitro* anther culture clustered in this group. When the number of sub-populations increased from two to three (*K* = 3) (Figure 2B), the population was sub-divided into wild, landraces, and anther culture accessions. The wild group consisted of the same 33 accessions identified in *K* = 2. The landrace group consisted of 42 accessions from Antioquia, Boyacá, Caldas, Cundinamarca, Nariño, and Norte de Santander, while the anther culture accessions consisted of 24 accessions mostly from Boyacá and Cundinamarca.

PCA was used to corroborate the sub-populations of the collection obtained by the Admixture software analysis. A three-dimensional scatter plot involving 99 accessions showed that the first three PCA axes accounted for 8.2, 2.3, and 2.0% of the genetic variation among populations, respectively. These results confirmed the separation of the accessions into three sub-populations: mostly wild, landraces and the accessions of anther culture (Figure 2C). The anther culture accessions were grouped in the PCA analysis probably because of the presence of homozygous loci that differentiate them from the landraces.

The NJ-based dendrogram showed that some accessions concurred with the geographical origin of the accessions (Figure 2D). The first group (in green) mostly contained wild accessions (47) from Nariño, Antioquia, Valle del Cauca, Boyacá, Cundinamarca, and Norte de Santander in Colombia, and the accessions from the international repositories. The second group (in red) contained 21 accessions, including the anther culture accessions and some landraces, such as the Colombian ecotype (14U424_2) and the accessions 09U292_2, 09U292_3, 09U293_2 09U295_1, and 09U296_1 from the Boyacá Department. The third group (violet) encompassed 23 accessions with a mixture of landraces from Boyacá, Cundinamarca, Antioquia, and some anther culture accessions. The fourth group (in blue) contained eight accessions with only landraces from Boyacá, Nariño, and Antioquia of Colombia, the main producing areas in the country.

Based on the F_ST_ values for the whole collection, we did not find a high level of differentiation between the wild and cultivated sub-populations (F_ST_ = 0.028). When wild, landraces, and anther culture sub-populations were compared based on pairwise F_ST_ values, the anther culture and wild populations were more distinct (0.044), followed by wild and landraces (0.032) while anther culture and landraces showed 0.031. The landraces maintained similar alleles in the wild population and the anther culture were derived from the landraces and maintained genetic similarity.

### Association analyses

The association analysis was carried out for 93 accessions because accession 09U039-1 presented low quality in the sequence data (mnSCov < 0.7). Besides, the accessions 12U398_1, 09U294_6, 12U366_1, 14U447_1, 14U425_1, and 09U295_4 presented a gametic chromosome number (*n* = 24 chromosomes) and the ploidy is positively correlated with fruit size according to Chevalier et al. (2014). On the other hand, the AM was carried out in 10 of the 18 traits because some traits were associated with the same SNP markers. For this reason, we grouped the traits as follows: FWI and FWII were combined in Fruit Weight (FW), WMH and MW were combined in Fruit Width (FWD), and HMW and MH were combined in Fruit Height (FH) (Table 5). In addition, the traits of fruit shape category (DI, FSI, FSII, OVO, and OBO) were not used for the association analysis because the genotypes were very homogenous for this character (93% presented ovoid fruit shape).

**Table 5:** Association statistics of most significantly associated loci with cape gooseberry traits using *K* = 2 and *K* = 3 sub-populations inferred by Admixture.

**Category**	**Traits**	**SNP position^+^**	**Ch**.	Locus^*^	**MAF**	***K*** = **2**		***K* = 3**		**Annotation**
						***P*-value**	**PV**	***P*-value**	**PV**	
Yield	Fruits weight per plant (FWP)	S03_52616353^a^	3	Solyc03g082690.2	0.13	1.00E-04	17,9	–	–	U-box domain-containing protein
		S11_33573667	11	Solyc11g043120.1	0.49	–	–	7.87E-05	17.8	Phosphatidylinositol Kinase
	Number of fruits (NF)	S02_55085162	2	Solyc02g094550.2	0.46	1.0E-04	18.1	1.01E-04	17.0	Gene of unknown function
		S02_55085163								
	Fruit Weight (FW)	S01_149166	1	Non-available	0.40	5.92E-05	18.8	9.67E-05	17.6	Gene of unknown function
		S02_44121109^b^	2	Solyc02g079590.2	0.48	3.80E-05	19.6	9.18E-05	17.7	Serine/threonine kinase receptor
		S03_52616353^a^	3	Solyc03g082690.2	0.13	9.70E-06	23.0	8.15E-06	23.6	U-box domain-containing protein
		S03_70268245^d^	3	Solyc03g123410.1	0.18	4.90E-05	19.0	2.15E-05	21.2	Oxalate oxidase-like germin
		S06_2049586^g^	6	Solyc06g008160.2	0.10	–	–	9.74E-05	17.5	Chloroplast FLU-like protein
		S11_1524907^k^	11	Solyc11g007040.1	0.49	5.11E-05	18.9	9.64E-05	17.3	Hydrolase alpha/beta fold family protein
		S12_3664835	12	Solyc12g010740.1	0.04	7.46E-05	18.0	9.94E-05	17.5	Receptor like kinase
		S12_6422882^l^	12	Solyc12g017230.1	0.50	5.33E-05	18.8	5.21E-05	19.0	Os04g0431000 protein
Fruit size	Fruit perimeter (FP)	S03_52616353^a^	3	Solyc03g082690.2	0.13	2.30E-05	21.1	3.94E-05	19.3	U-box domain-containing protein
		S05_11429752	5	Non-available	0.30	8.40E-05	18.0	–	–	Gene of unknown function
		S11_1447930^j^	11	Solyc11g006940.1	0.40	2.40E-05	21.0	4.01E-05	19.3	Pentatricopeptide repeat-containing protein
		S11_1447931^j^								
		S11_1447966^j^	11							
		S11_1524907^k^	11	Solyc11g007040.1	0.49	7.90E-05	18.1	–	–	Hydrolase alpha/beta fold family protein
	Fruit area (FA)	S02_44121109^b^	2	Solyc02g079590.2	0.48	8.70E-05	17.7	–	–	Serine/threonine kinase receptor
		S03_52616353^a^	3	Solyc03g082690.2	0.13	1.90E-05	21.4	3.15E-05	19.8	U-box domain-containing protein
		S03_63746646^c^	3	Solyc03g113680.2	0.16	2.70E-05	20.5	2.44E-05	20.4	Microtubule-associated protein TORTIFOLIA1
		S03_70268245^d^	3	Solyc03g123410.1	0.18	8.40E-05	17.8	7.57E-05	17.7	Oxalate oxidase-like germin 171
		S04_4375270^e^	4	Solyc04g012040.2	0.25	4.60E-06	19.2	5.01E-05	18.7	Proteosoma 26S
		S05_1948424^f^	5	Solyc05g007290.1	0.11	8.00E-05	17.9	7.45E-05	17.8	Regulator of chromosome condensation domain-containing protein
		S06_2049586^g^	6	Solyc06g008160.2	0.10	6.30E-05	18.5	5.26E-05	18.6	Chloroplast FLU-like protein
		S09_17003764^h^	9	Solyc09g018790.2	0.17	8.10E-05	17.9	9.33E-05	17.2	Gamma hydroxybutyrate dehydrogenase-like protein
		S10_62358117^i^	10	Solyc10g081250.1	0.23	7.20E-05	18.1	4.05E-05	19.2	DNA polymerase
		S11_1447930^j^	11	Solyc11g006940.1	0.40	9.50E-05	17.5	–	–	Pentatricopeptide repeat-containing protein
		S11_1447931^j^								
		S11_1447966^j^								
	Fruit width (FWD)	S02_43088856	2	Solyc02g078390.2	0.40	6.20E-05	18.7	–	–	MutS2 protein
		S02_43089010			0.39	7.80E-05	18.1	–	–	
		S02_43089036								
		S02_55241693	2	Solyc02g094640.2	0.44	5.80E-05	18.9	–	–	Acetyl-coenzyme A synthetase
		S03_52616353^a^	3	Solyc03g082690.2	0.13	2.23E-05	21.2	3.71E-05	19.8	U-box domain-containing protein
		S03_63746646^c^	3	Solyc03g113680.2	0.16	2.20E-05	21.2	3.29E-05	20.1	Microtubule-associated protein TORTIFOLIA1
		S03_70268245^d^	3	Solyc03g123410.1	0.18	3.20E-05	20.3	3.64E-05	19.9	Oxalate oxidase-like germin 171
		S04_4375270^e^	4	Solyc04g012040.2	0.25	2.60E-05	20.8	2.07E-05	21.3	26S proteasome non-ATPase regulatory subunit 12
		S05_1948424^f^	5	Solyc05g007290.1	0.11	4.10E-05	19.7	6.16E-05	18.6	Regulator of chromosome condensation domain-containing protein
		S06_2049586^g^	6	Solyc06g008160.2	0.10	2.10E-05	21.4	2.31E-05	21.0	Chloroplast FLU-like protein
		S09_17003764^h^	9	Solyc09g018790.2	0.17	2.20E-05	21.2	3.85E-05	19.7	Gamma hydroxybutyrate dehydrogenase-like protein
		S10_62358117^i^	10	Solyc10g081250.1	0.23	4.30E-05	19.6	3.96E-05	19.7	DNA polymerase
		S11_1447930^j^	11	Solyc11g006940.1	0.40	9.10E-05	17.8	–	–	Pentatricopeptide repeat-containing protein
		S11_1447931^j^								
		S11_1447966^j^								
		S12_6422882^l^	12	Solyc12g017230.1	0.50	8.90E-05	17.8	9.18E-05	17.7	Os04g0431000 protein
	Fruit height (FH)	S03_52616353^a^	3	Solyc03g082690.2	0.13	8.30E-05	17.7	9.75E-05	16.8	U-box domain-containing protein
		S03_63746646^c^	3	Solyc03g113680.2	0.16	2.30E-05	20.8	2.15E-05	20.5	Microtubule-associated protein TORTIFOLIA1
		S10_62358117^i^	10	Solyc10g081250.1	0.23	9.90E-05	17.3	5.85E-05	18.1	DNA polymerase
Fruit quality	Cracked fruits percentage (CF)	S06_45033846	6	Solyc06g073100.2	0.46	9.80E-05	15.2	–	–	GDSL esterase/lipase At3g27950
		S06_45033861								
		S06_49262147	6	Nonavailable	0.49	8.40E-05	15.6	1.95E-05	18.9	Gene of unknown function
	Firmness (FIR)	S07_57560621	7	Solyc07g043610.2	0.02	6.60E-05	18.4	5.86E-05	18.0	Auxin response factor 6
		S08_51945090	8	Nonavailable	0.19	3.57E-05	19.9	7.74E-05	17.3	Gene of unknown function
		S08_51945138								
		S11_4540005	11	Solyc11g011490.1	0.02	6.60E-05	18.4	5.86E-05	18.0	Gene of unknown function
		S11_4540016								
	Soluble solids concentration (SST)	S01_66177533	1	Nonavailable	0.38	7.70E-05	16.3	–	–	Gene of unknown function
		S06_43666281	6	Solyc06g071080.2	0.41	–	–	1.37E-04	12.5	Proton-dependent oligopeptide transport family protein

+ *SNP genomic position on the tomato reference genome. Ch., Chromosome*.

* *Name of the locus to which the peak SNP belongs (according to the tomato genome annotation version 2.50). PV (phenotypic variability explained) and MAF (minimum allele frequency) are given*.

a, b, c, d, e, f, g, h, I, j, k, l *Associated SNPs for multiple traits*.

Analysis of LD decay was not carried out because the reference genome was unavailable for the species. The LD was estimated using the squared correlation (*r*^2^) from pairs of all SNP markers without the LD filter being specified and using the tomato genome as a reference. A total of 38,884 pairs of markers showed a significant LD value with an average of 0.008 and, from these, 13,184 pairs of markers showed an *r*^2^ ≤ 0.01.

**Figure 3:**
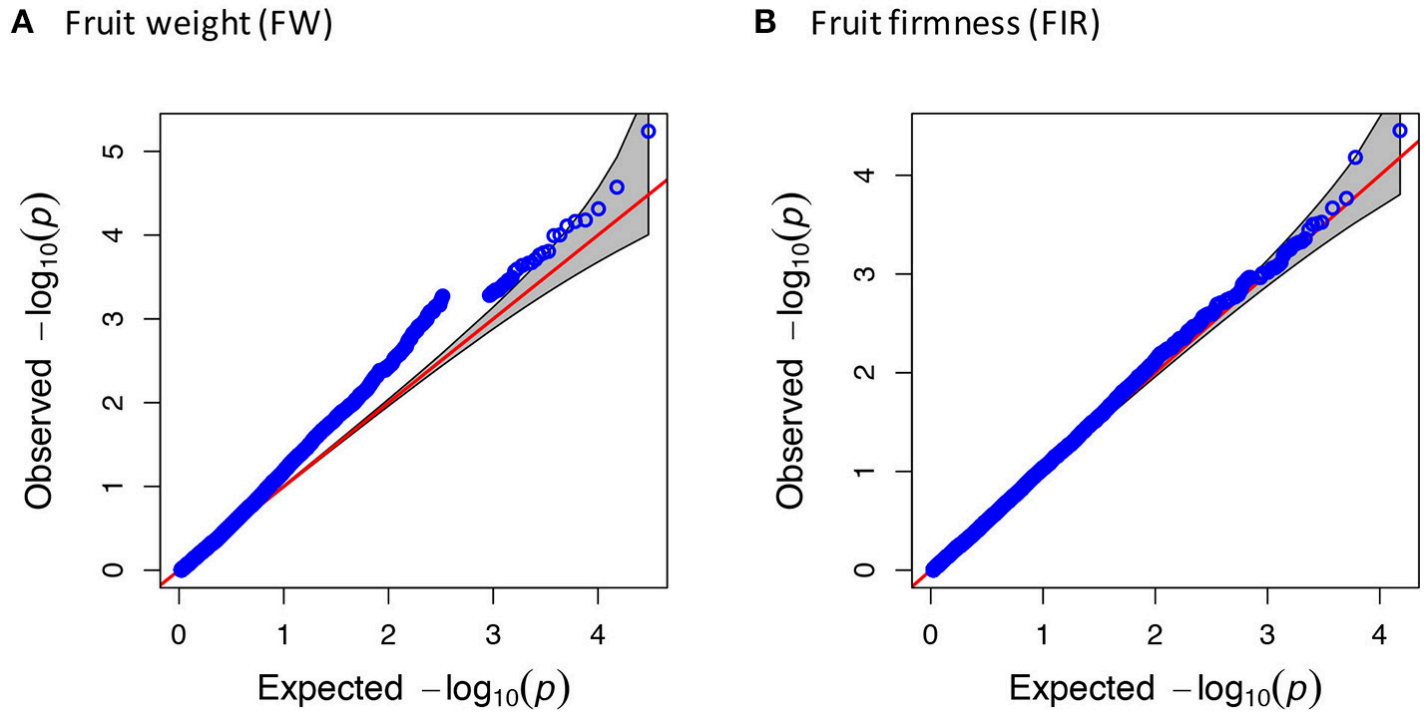
Quantile-quantile (Q-Q) plot for fruit weight and fruit firmness. Q-Q plots showing the ratio of the observed *p*-values (blue dots) compared to the expected *p*-value distribution (red line) for **(A)** fruit weight and **(B)** fruit firmness.

**Figure 4:**
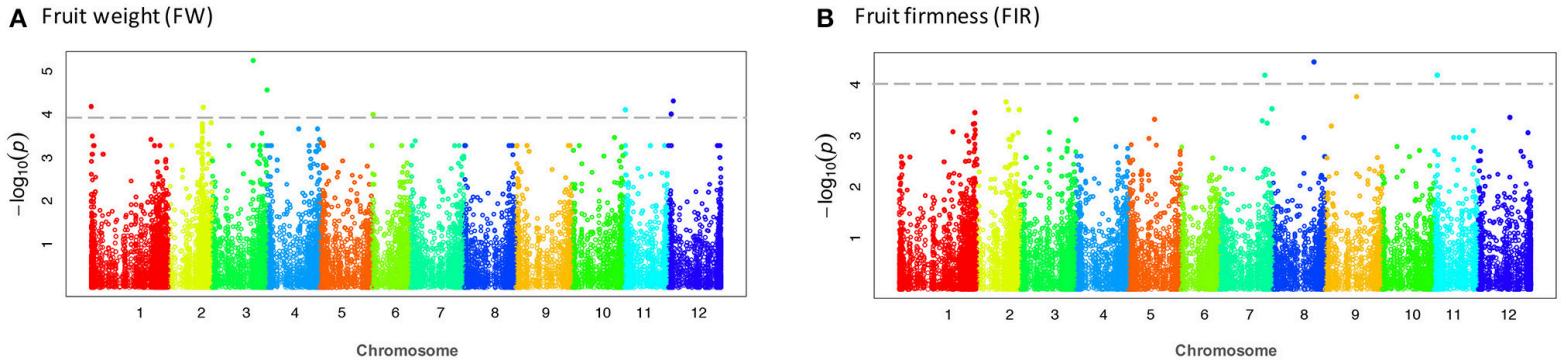
Manhattan plots showing significant associations for fruit weight and fruit firmness. Chromosome number are displayed along the X-axis and the negative log_10_ of the association *p*-value for each SNP on the Y-axis. Higher negative log_10_ indicates stronger association with the trait. **(A)** fruit weight and **(B)** fruit firmness.

The CP and FH traits that displayed non-normal distribution were transformed with the Box-Cox transformation procedure to improve sensitivity and to avoid false positives in small sample sizes, according to Goh and Yap (2009). According to the kinship analysis based on the Loiselle logarithm, the accessions were unrelated. Using a significance threshold of –log_10_(*p*) ≥ 4.0, after the FDR correction, we did not identify any significant association. Considering this aspect, we reduced the threshold parameter to LOD Score = 4.0, supported by the Q-Q plots that evidenced the association of the SNP-trait with lower but still significant *p*-values (*p* ≤ 1.0E-04) before the FDR correction (Figure 3, Figure S2). The significant associations detected by the MLM were visualized in a Manhattan plot (Figure 4, Figure S3).

Using the Q-matrix for both *K* = 2 and *K* = 3, we found 34 unique SNPs, which mapped to 21 distinct tomato genes with *p*-values ≤ 1.0E-04 (Table 5). The largest association numbers were detected for the FW, FA, and FWD with 7, 10, and 12 associated markers, respectively. For yield traits, 10 SNP markers were located inside nine genes and one SNP (S01_149166) was located nearby of Solyc01g005190.1 gene. These markers explained between 17.0 and 23.6% of the phenotypic variation and were located on chromosomes 1, 2, 3, 6, 11, and 12 in the tomato. For the fruit size traits, 19 SNP markers were identified that explained between 16.8 and 21.3% of the phenotypic variation and were located inside 14 genes on chromosomes 2, 3, 4, 5, 6, 9, 10, 11, and 12. Finally, a total of 10 SNP markers located in four genes were identified as significantly associated with fruit quality. These markers explained between 12.5 to 19.9% of the phenotypic variation and were located on chromosomes 1, 6, 7, 8, and 11 (Table 5).

Some phenotype/genotype associations were related to multiple traits. For *K* = 2, four markers: S02_44121109 (Solyc02g079590.2), S03_70268245 (Solyc03g123410.1), S11_1524907 (Solyc11g007040.1), and S12_6422882 (Solyc12g017230.1) showed an association with both fruit weight and fruit size. Marker S03_52616353 (Solyc03g082690.2) was associated with three characteristics: fruit weight per plant (FWP), fruit weight and fruit size (as area, perimeter, height, and width). For *K* = 3, the SNP markers S03_70268245, S06_2049586 (Solyc06g008160.2), and S12_6422882 showed association with the fruit weight and fruit size, as did marker S03_52616353, but with no significant association with the FWP.

## Discussion

Highly significant differences (*p* ≤ 0.0001) were observed between the accessions for all of the studied traits (Table 1). These results agree with the conclusions of Herrera et al. (2011) who reported significant differences for yield and the average weight of fruit with calyx (*p* ≤ 0.0001). Earlier studies failed to identify significant differences in the number of fruits per plant although this observation was probably due to differences in the number of accessions evaluated. The fruit shape traits (DI, FSI, FSII, OVO, and OBO) showed no significant differences between the replications, probably suggesting that fruit shape was not greatly influenced by environmental factors. Similar results have been reported by Liu et al. (2017) in the FSI of the tomato. The CF and FIR also showed no significant differences between replications, but significant differences between accessions, suggesting a genetic effect contribution to these traits, as has been demonstrated in the tomato (Mustafa et al., 2017). The development of cracking-resistant varieties can be an effective solution for the cape gooseberry.

The coefficient of variation for the major traits ranged from 3.47 to 10.54% for traits related to fruit size and weight. The high variability observed in the FWP and NF is similar to the findings of Herrera Moreno et al. (2012) and could be explained by the relationship between yield and other variables such as length and the number of internodes in productive shoots. Fruit cracking was the most variable trait between accessions, possibly due to differences in the shape and arrangement of sub-epidermis cells of the fruits of the different accessions, as reported for the sweet cherry (Demirsoy and Demirsoy, 2004), or cuticular membrane thickness, as reported for the tomato (Matas et al., 2004).

The more important correlations were CF-FWP, CF-FWI-FWII, and CF-OVO, which showed a strong negative relationship between the cracking and size, weight, and asymmetry of the fruits. This observation suggests that the phenomenon of fruit cracking may not result from quick filling. Not all of the accessions showed the same percentage of fruit cracking as has been reported by Herrera et al. (2011) who reported differences in the percentage of cracking in 54 accessions of cape gooseberry. Our results supported the hypothesis that cracking in the cape gooseberry involves a genetic component and genetic variation that may permit breeding progress as suggested by Cooman et al. (2005).

According to Singh (2001), the heritability of FWP and NF were medium and moderately high, respectively, and the other traits were very high. This high heritability indicates a small contribution of environmental factors to the phenotype. High to medium estimates of broad sense heritability have also been reported by Leiva-Brondo et al. (2001) for yield, fruit weight, fruit shape (length/width), soluble solids content, titratable acidity, and ascorbic acid content.

The PCA clustering for the first four principal components explained 89.87% of total variance. The weight and size traits and fruit firmness and cracking percentage showed the highest contribution. Similar results were found in the earlier analysis of Herrera Moreno et al. (2012) who reported that the first four PCs explained 70.19% of the variance and were related to the physical aspects of fruits, such as weight, volume, and diameter. Similar results were found by Morillo et al. (2011) who reported that the first three PCs explained 81.75% of the variance and were represented by measures of fruit size. In this study, we identified numerous wild and cultivated accessions with desirable horticultural characteristics, such as high yield and fruit quality, highlighting seven accessions with less than 4% cracking fruit percentage and high yield that have been included in breeding programs for developing varieties from recurrent selection schemes.

The accessions 12U398_1, 09U294_6, 12U366_1, 14U447_1, 14U425_1, and 09U295_4 showed the lowest FW, haploid accessions with *n* = 24 chromosomes and ploidy that can affect the fruit size (Chevalier et al., 2014), explaining these results. In contrast, the accessions that have shown the highest values are in foreign and previous studies, reporting that African accessions produce bigger fruits than Colombian ones (Fischer et al., 2007). The accessions 09U033_1 and 09U277_5 showed the highest FWP and NF, indicating that the FWP was influenced by the number of fruit and not by fruit weight.

We reported the identification of 27,982 SNPs in the cape gooseberry using the software Tassel v5.0. Enhanced SNP discovery, SNP quality, and production steps and some optimization of parameters improved the SNP detection over the 1,739 SNPs previously reported (Osorio-Guarín et al., 2016). Based on the SNPs identified, the mean expected heterozygosity value was lower than the observed heterozygosity, indicating an excess of heterozygotes probably because of high rates of cross-pollination of the species, around 54% (Lagos et al., 2008). Our results are similar to the study published by Berdugo et al. (2015) who reported values of H_E_ = 0.44, H_O_ = 0.73, and PIC = 0.35 for a collection of parents, intra, and interspecific hybrids for *P. peruviana* and *P. floridana* evaluated with COSII and IRGs markers. Similarly, Garzón-martínez et al. (2015), using 47 *P. peruviana* accessions analyzed with SNP markers, found a mean value of H_E_ = 0.41 and PIC = 0.32. However, Garzón-martínez et al. (2015) reported a value of H_O_ = 0.59 and Osorio-Guarín et al. (2016) reported diversity values of H_E_ = 0.665, H_O_ = 0.431, and PIC = 0.344 using 100 accessions analyzed with 1,739 SNP markers. The discrepancies could be due to differences in the population studied and the use of a low number of SNP markers compared with this study.

Again, the variability in the collection and markers used likely explains the slight differences compared with this study. In general, the collection showed a low genetic differentiation, possibly because of allogamy of the species as reported by Silvertown and Charlesworth (2009). Low differentiation, high genetic variation, and an excess of heterozygotes are characteristics of outbreeding populations. Our results are similar to those reported by Garzón-martínez et al. (2015) who found F_ST_ (0.038) values for *P. peruviana* and related taxa populations using SNP markers. Likewise, Chacón et al. (2016) found a low genetic differentiation (F_ST_ = 0.058) between cultivated and non-cultivated populations using SSR markers in 345 cape gooseberry accessions. In contrast, Osorio-Guarín et al. (2016) found a high F_ST_ value (0.3507) when analyzing 100 accessions with SNP markers. This strong discrepancy may be explained by the differences in accessions used and the number of loci analyzed.

The overall level of detected LD was low, which indicates high recombination, as expected in allogamous and partially allogamous species (Rafalski and Morgante, 2004). Estimates of cross-pollination in *P. peruviana* exceed 52%, which would support rapid LD decay (Rafalski, 2010). A total of 34 marker-trait associations were identified. Many associations with the FW, FA, and FWD were found but only two associations for the FWP, NF, and SST. FWP is a quantitatively inherited trait and it tends to correlate with the number of fruits produced by each plant and fruit weight. For yield, the tomato gene Solyc03g082690.2, associated with FWP and FW, is related to the U-box domain-containing protein involved in cellular processes, including cell cycle regulation, vesicle-mediated protein transport, protein folding, and protein degradation (Azevedo et al., 2001). The gene Solyc01g005190.1, associated with the FW, is related to the zinc fingers protein involved in early fruit development, as reported in the tomato (Aiese Cigliano et al., 2013) and nicotiana fruit (Wu et al., 2014).

The associations identified for the fruit size traits were distributed over nine chromosomes (no associations detected on chromosomes 1, 7, and 8 chromosomes). The gene Solyc04g012040.2, associated with the FWD and FA, is annotated as a 26S proteasome, involved in protein degradation and the balancing of cell expansion with cell proliferation rates as has been reported by Kurepa et al. (2009) in Arabidopsis. The gene Solyc09g018790.2, associated with both FWD and FA, is annotated as a Gamma hydroxybutyrate dehydrogenase-like protein, which is involved in multiple physiological responses and plays an important role during early fruit development in the tomato (Fait et al., 2008; Takayama and Ezura, 2015). Furthermore, the gene Solyc11g006940.1, coding a pentatricopeptide repeat protein, is involved in plant growth and development (Sharma and Pandey, 2015).

In general, fruits with an ovoid shape are more desirable and a higher FWP and lower CF. FIR and CF are important for increasing the shelf-life of this fruits and preventing the early appearance of fungal and bacterial diseases that alter the organoleptic characteristics. For fruit cracking, we found an association with the gene Solyc06g073100.2 which is annotated as a lipase and esterase enzyme involved in the deposition of the cutin polyester in the tomato fruit cuticle (Girard et al., 2012). In contrast, the fruit firmness was associated with the gene Solyc07g043610.2, which is annotated as an auxin response factor involved in the control of the ripening process and fruit firmness in the tomato (Hao et al., 2015; Breitel et al., 2016). For the SST, the gene Solyc06g071080.2 is related to the proton-dependent oligopeptide transport family protein involved as a nitrate transporter (Tsay et al., 2007). It is tempting to think of these associations as causal though further research would be needed to establish such a relationship.

Co-localized associations for the FWP, FW, FA, FP, FH, and FWD were identified. Such co-localization might be related to the pleiotropic effects of the genes or result from genetic linkage. Similar results have been reported in the tomato for fruit traits such as soluble solids and sugar content, titratable acidity, fruit weight, and locule number (Xu et al., 2013), in rice for flowering and yield (Zhao et al., 2011) and in the cape gooseberry for response to *Fusarium oxysporum* (Osorio-Guarín et al., 2016). Based on the high synteny reported between members of the Solanaceae family (Wang et al., 2008), we assume that the function of some genes found in the tomato should be conserved in the cape gooseberry. The detected associated markers could be then recommended for fruit yield and size improvement in cape gooseberry breeding programs after functional confirmation.

## Conclusions

The association mapping population used in this study presented high phenotypic and genetic variability that can be exploited in plant breeding programs. The results allowed for the identification of promising material for breeding programs with a high FWP and low CF. Fruit cracking and lack of firmness might be related to genetic events since the results showed clear differences among the accessions. This could be useful for exhaustive studies on the heritability and genetic architecture of these traits in breeding. Our findings suggested that using SNP markers and the mixed linear model were suitable for detecting significant associations and allowed for the detection of 34 associations for the main cape gooseberry fruit traits. The important correlation of the FH and FWD on FWP and the co-location of one associated SNP suggest that fruit size SNPs can have a strong effect on the yield of the cape gooseberry. Furthermore, novel SNP markers for yield (FWP, NF, and FW), fruit size (FP, FA, FWD, and FH), and fruit quality (FIR, CF, and SST) were found, and it should be noted that this study is an important contribution to the knowledge on the genetic basis of some traits in the cape gooseberry.

## Author contributions

VN conceived the study. FG-A and JO-G analyzed the data. FG-A prepared the manuscript and JO-G edited. All authors read and approved the final version of the manuscript.

### Conflict of interest statement

The authors declare that the research was conducted in the absence of any commercial or financial relationships that could be construed as a potential conflict of interest.
