# Supplementary figures and images for: Association Study Reveals Novel Genes Related to Yield and Quality of Fruit in Cape Gooseberry (Physalis peruviana L.)
Source: Front Plant Sci. 2018 Mar 20;9:362. doi: 10.3389/fpls.2018.00362 (PMC5869928; doi:10.3389/fpls.2018.00362)

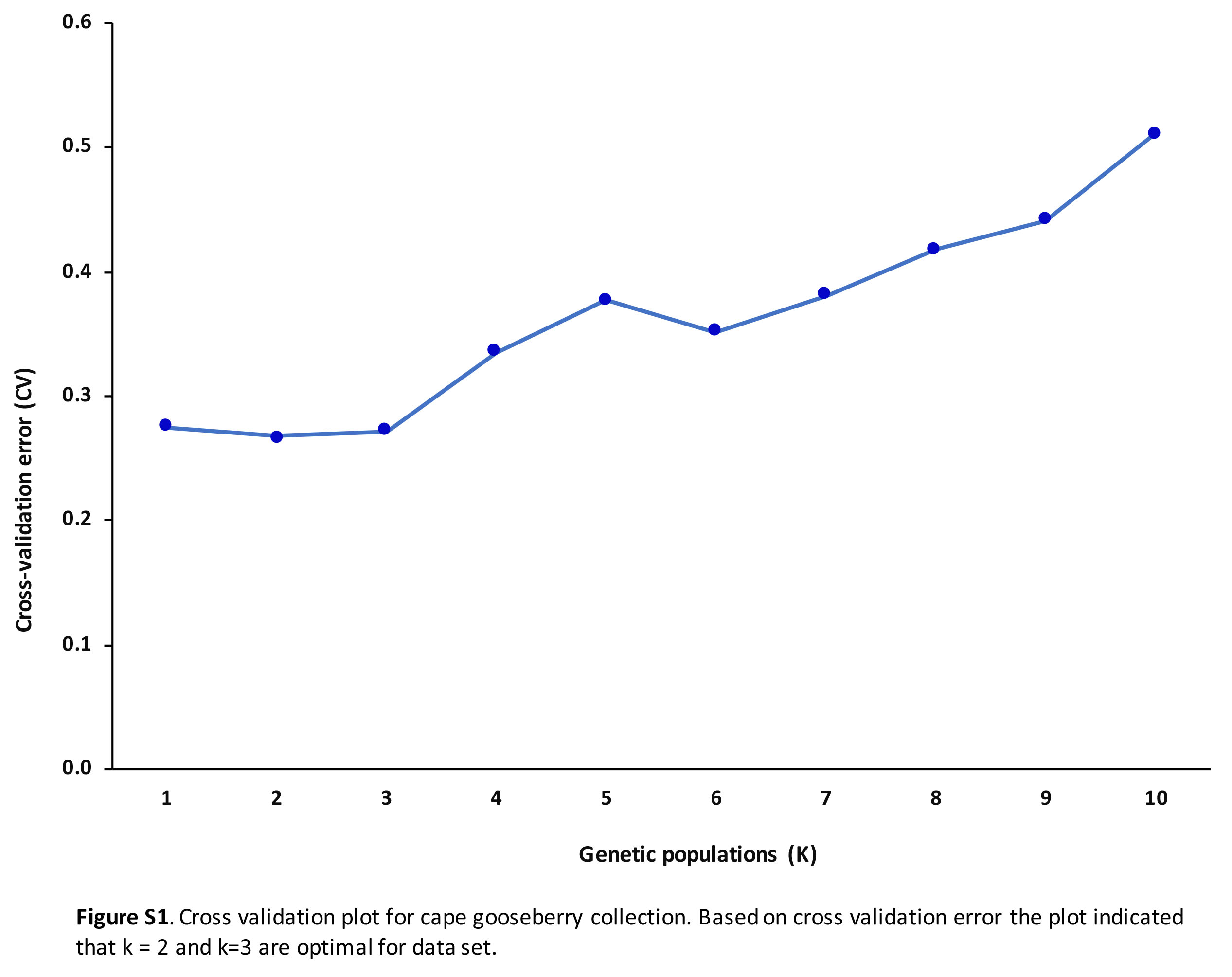

Supplement: Supplementary file 4 [file Image1.JPEG]

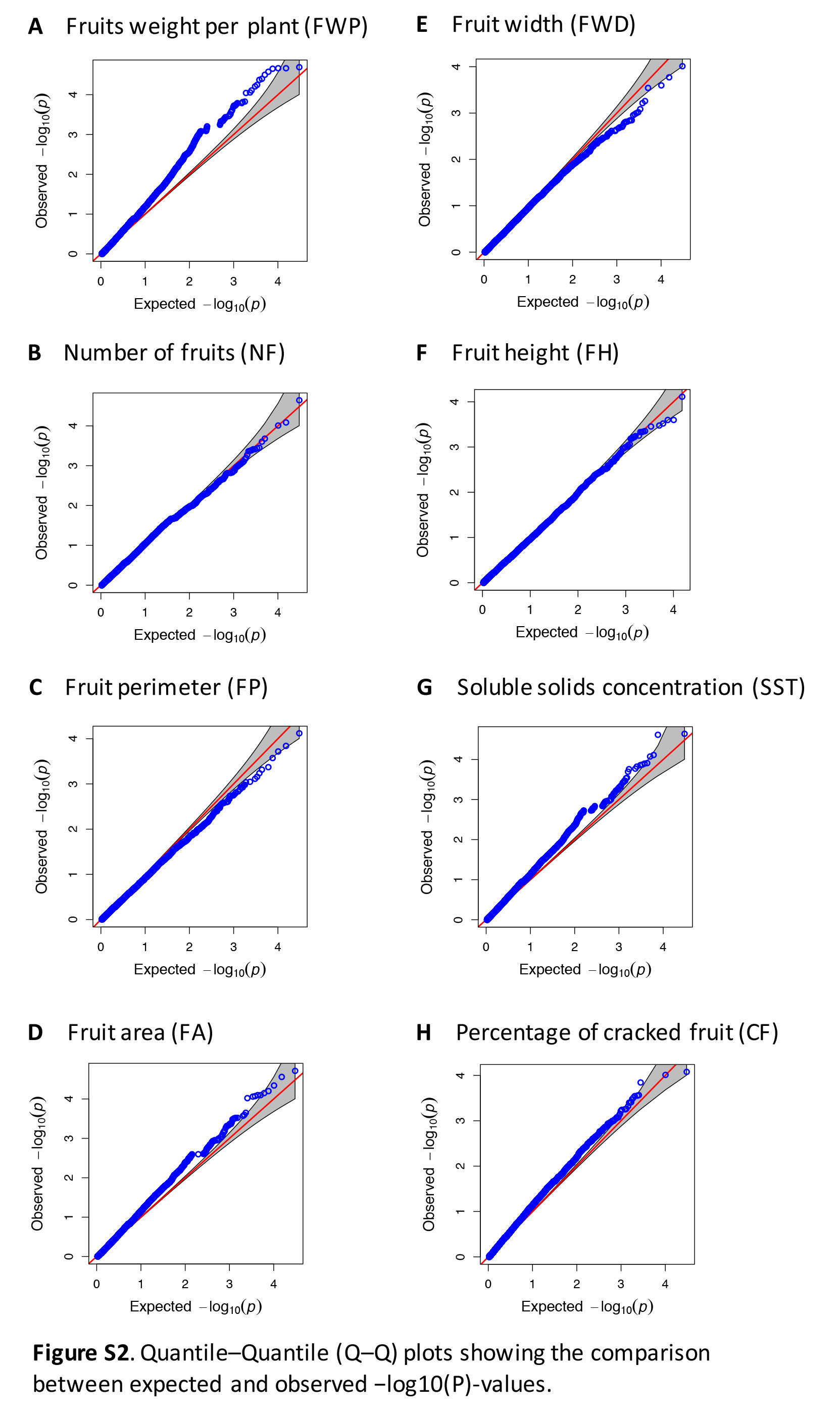

Supplement: Supplementary file 5 [file Image2.JPEG]

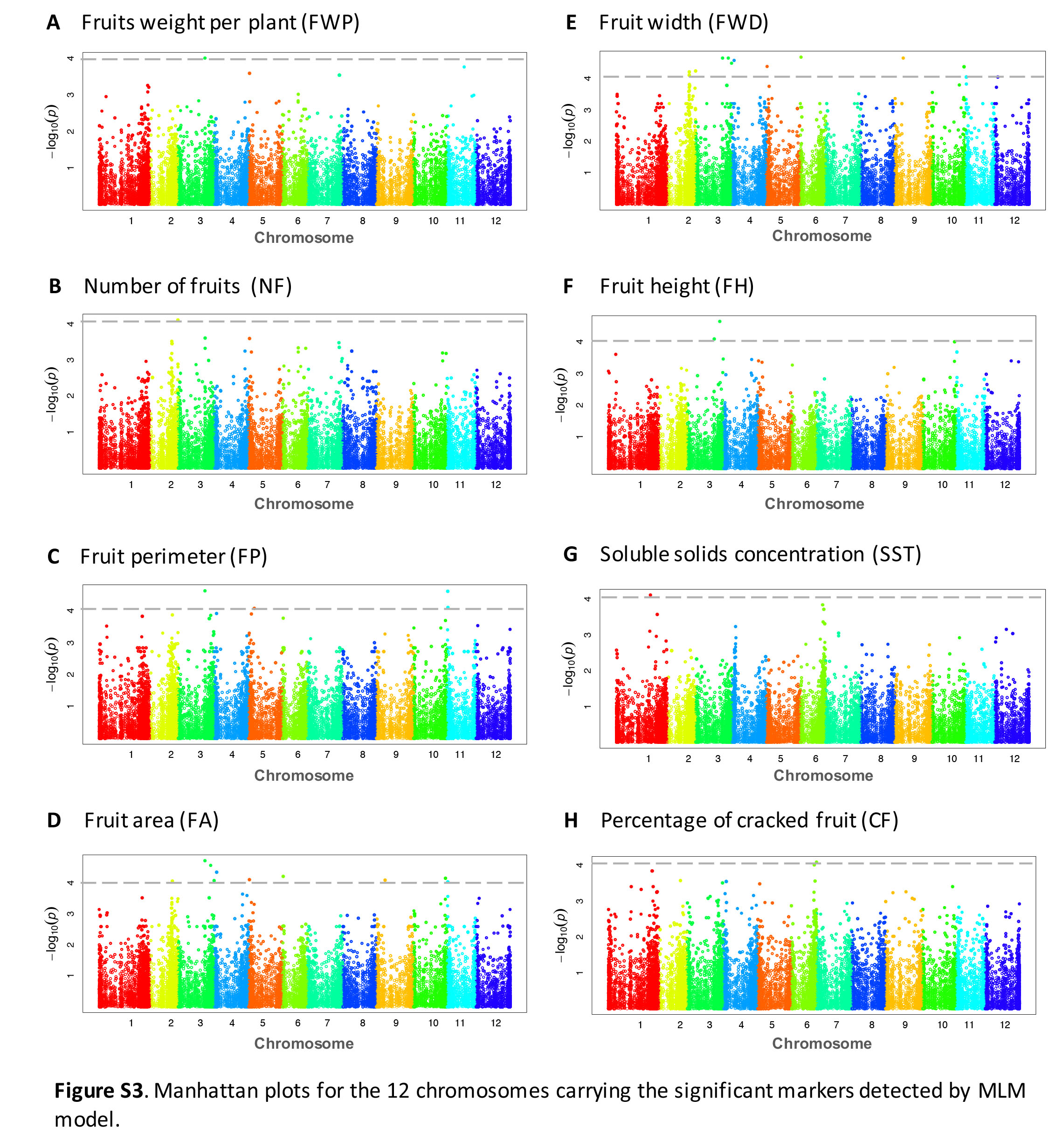

Supplement: Supplementary file 6 [file Image3.JPEG]
